# Qushi huayu decoction dose-dependent inhibition of caspase-2/SREBP-1 in MASLD mice

**DOI:** 10.1186/s41065-025-00561-x

**Published:** 2025-09-29

**Authors:** Qian Liu, Zuxi Gu, Xin Xin, Xiaoping Shen, Xiaojun Gou, Lixin Hou, Shuang Li

**Affiliations:** 1 Department of Gastroenterology, Baoshan Hospital Affiliated to Shanghai University of Traditional Chinese Medicine, No. 181, Youyi Road, Baoshan District, Shanghai, 201999 China; 2 Laboratory Animal Facility, Science and Technology Experimental Center, Shanghai University of Traditional Chinese Medicine, Shanghai, 201203 China; 3 Institute of Liver Diseases, Shuguang Hospital, Shanghai University of Traditional Chinese Medicine, Shanghai, 201203 China; 4 Central Laboratory, Baoshan Hospital, Shanghai University of Traditional Chinese Medicine, Shanghai, 201999 China; 5 Luodian Hospital, Baoshan District, Shanghai, 201908 China

**Keywords:** Qushi huayu decoction, Metabolic dysfunction-associated fatty liver disease, Caspase-2/SREBP-1 signaling pathway

## Abstract

**Background:**

Metabolic Dysfunction-Associated Steatotic Liver Disease (MASLD) is characterized by hepatic lipid accumulation and metabolic disturbances. Caspase-2 cleaves site-1 protease (S1P), leading to the persistent activation of sterol regulatory element-binding proteins (SREBPs), which subsequently promote the progression of MASLD. Previous studies have demonstrated that the Qushi Huayu Decoction (QHD) significantly alleviates MASLD, particularly inhibiting the expression of SREBP-1 in hepatocytes of MASLD mouse models. However, its regulatory effect on the Caspase-2/SREBP-1 pathway and the dose-dependent nature of these effects remain unclear.

**Objective:**

The regulatory effects of high, medium, and low doses of Qushi Huayu Decoction (QHD) on the Caspase-2/SREBP-1 pathway and their potential dose-dependent impacts was investigated.

**Method:**

A MASLD model was induced in 28-week-old C57BL/6J mice using a high-fat diet (HFD). Mice were treated with QHD granules at high (3.466 g/kg), medium (1.733 g/kg), and low doses (0.867 g/kg), as well as a Caspase-2 inhibitor for a duration of 5 weeks. Pharmacodynamic indicators, including triglycerides (TG) and free fatty acids (FFA) in liver tissue, hepatic histopathology, and serum biochemical markers, were assessed. The expression of genes in the Caspase-2/SREBP-1 signaling pathway and its downstream targets was also analyzed.

**Results:**

QHD at all doses effectively improved hepatic steatosis. The low-dose group significantly reduced hepatic TG levels (*p* < 0.01) and the insulin resistance index (*p* < 0.05). The high-dose group significantly inhibited the expression of Caspase-2 protein (*p* < 0.01) and nuclear SREBP-1 protein (*p* < 0.05), with a dose-dependent decrease in Caspase-2 activity.

**Conclusion:**

QHD exhibits dose-dependent, complementary effects in MASLD, with low doses improving lipid metabolism and insulin sensitivity, and high doses more effectively suppressing Caspase-2/SREBP-1 and inflammatory signaling. This dual action underscores its broad regulation of ER stress and supports stage-specific, hierarchical dosing strategies aligned with traditional Chinese medicine principles.

**Supplementary Information:**

The online version contains supplementary material available at 10.1186/s41065-025-00561-x.

## Introduction

In 2023, an international consensus recommended renaming non-alcoholic fatty liver disease (NAFLD) to metabolic dysfunction-associated steatotic liver disease (MASLD), emphasizing its inseparable relationship with metabolic syndrome, including obesity, insulin resistance, and dyslipidemia [1]. ​The core pathological feature of MASLD is hepatocellular lipid deposition (fatty change ≥ 5%), accompanied by at least one metabolic disorder indicator, such as increased waist circumference, elevated blood glucose levels, or hypertension [2]. Lipid metabolism imbalance is the core mechanism driving the progression of MASLD. Endoplasmic reticulum (ER) stress is intricately and significantly linked to lipid synthesis and metabolic liver diseases (MASLD) [3]. ER stress promotes de novo lipogenesis (DNL) by activating the unfolded protein response (UPR) and the SREBP-1 pathway. This activation can trigger a vicious cycle of “lipotoxicity-ER stress” [4]– [5]. Recent studies indicate that ER stress, induced Caspase-2 can cleave site-1 protease (S1P), generating an active fragment that initiates SCAP-independent SREBP activation, thereby promoting hepatic lipid accumulation [6]. In this context, the IRE1α/Caspase-2-S1P signaling branch has been implicated in SREBP-1 activation and may exacerbate the “lipotoxicity–inflammation” feedback loop, highlighting its potential as a therapeutic target in MASLD [6, 7].

Most research on MASLD treatment has focused on single-target inhibitors [8]– [9]. However, complete inhibition of ER stress may impair cellular adaptability [3]– [10]. Moreover, single-target drugs, such as the acetyl-CoA carboxylase (ACC) inhibitor NDI 010976, often fail because compensatory lipid uptake increases [8]. In contrast, the multi-target nature of traditional Chinese medicine (TCM) formulas offers a new strategy for MASLD by dynamically balancing the ER stress network through multidimensional regulation of “metabolism-lipid synthesis-inflammation” [11]– [12].

Qushi Huayu Decoction (QHD) is a traditional Chinese medicine formula that has been clinically validated as an effective treatment for metabolic dysfunction-associated fatty liver disease (MASLD) [13]– [14]. Several studies have revealed multiple mechanisms by which QHD acts. First, it regulates liver lipid metabolism. QHD can reduce hepatic lipid accumulation by modulating liver fat metabolism [15]. For instance, it inhibits lipid accumulation via activation of the AMPK pathway [16]. Tian et al. further showed that QHD improves fructose-induced liver steatosis by suppressing XBP1s-driven lipogenesis [17]. Second, QHD regulates the gut microbiota. It has been found that QHD can regulate the gut microbiota and improve intestinal barrier function, thereby alleviating non-alcoholic steatohepatitis (NASH) [18]– [19]. Leng et al. found that the improvement of NASH by QHD is associated with the inhibition of the mitogen-activated protein kinase (MAPK) pathway in the gut [18]. Third, QHD promotes fatty acid β-oxidation. It has been shown by Sun et al. that QHD reduces hepatic lipid accumulation through the JAK2/STAT3/CPT-1 A-related fatty acid β-oxidation pathway, thus treating non-alcoholic steatohepatitis (NASH) [15]. Fourth, QHD regulates metabolism. Metabolomics studies have indicated that QHD can regulate metabolic disorders in rats with fatty liver induced by a high-fat diet [20]– [21]. Gou et al. used metabolomics to investigate the effects of QHD on high-fat diet-induced fatty liver in rats and found that QHD improves fatty liver [20]. Fifth, QHD exerts multi-target therapeutic mechanisms. It has been demonstrated by Feng et al. that QHD, or its active ingredients (such as geniposide and chlorogenic acid, GC), influence the hepatic transcriptome and gut microbiota in NAFLD rats. These effects include increasing the expression of genes necessary for glutathione production and decreasing the expression of genes involved in lipid synthesis [14].

The multi-target effects of QHD make it a promising candidate for the treatment of MASLD. However, the specific mechanisms of action of QHD still require further investigation. It remains unclear whether QHD targets the Caspase-2/SREBP-1 pathway, and whether its multi-component nature leads to dose-dependent variations in its effects. To address these questions, this study employed a 28-week high-fat diet (HFD) induced C57BL/6J mouse model to simulate chronic metabolic stress in humans [22]. The inhibitory effects of different doses of QHD on the Caspase-2/SREBP-1 pathway were observed.

## Materials and methods

### Drug preparation

Qushi Huayu Decoction (QHD) is a traditional Chinese medicine formula consisting of five herbs: *Gardenia jasminoides Ellis*. (Zhi Zi), *Artemisia capillaries Thunb.* (Yin Chen), *Hypericum japonicum Thunb*. (Tian Ji Huang), *Polygonum cuspidatum Sieb. et Zucc*. (Hu Zhang), and *Curcuma longa L*. (Jiang Huang). The granules are produced by Jiangyin Tianjiang Pharmaceutical Co., Ltd. (Batch No. 20184Y0245), with a clinical dosage of 5.2 g, administered twice daily. The quality control is based on the main active ingredients, which were detailed in a previous study published [18]. The mouse dosage was derived from the human equivalent dose using the body surface area (BSA) normalization method [23]. Based on the clinical dose (5.2 g × 2 = 10.4 g/day for a 60-kg adult, equivalent to 0.173 g/kg), the calculated mouse equivalent dose was 1.733 g/kg. This was defined as the medium dose. To evaluate potential dose-dependent effects, a low dose (0.867 g/kg, 0.5× equivalent) and a high dose (3.466 g/kg, 2× equivalent) were also included. This gradient design is consistent with a patented methodology for QHD dosage optimization (Patent No. ZL200610009140.0.0, CNIPA), providing a standardized basis for the selection of low, medium, and high doses. The Caspase-2 inhibitor Ac-VDVAD-CHO (Catalog No. 12352200) was purchased from Millipore Sigma.

### Animal model and grouping

**Fig. 1:**
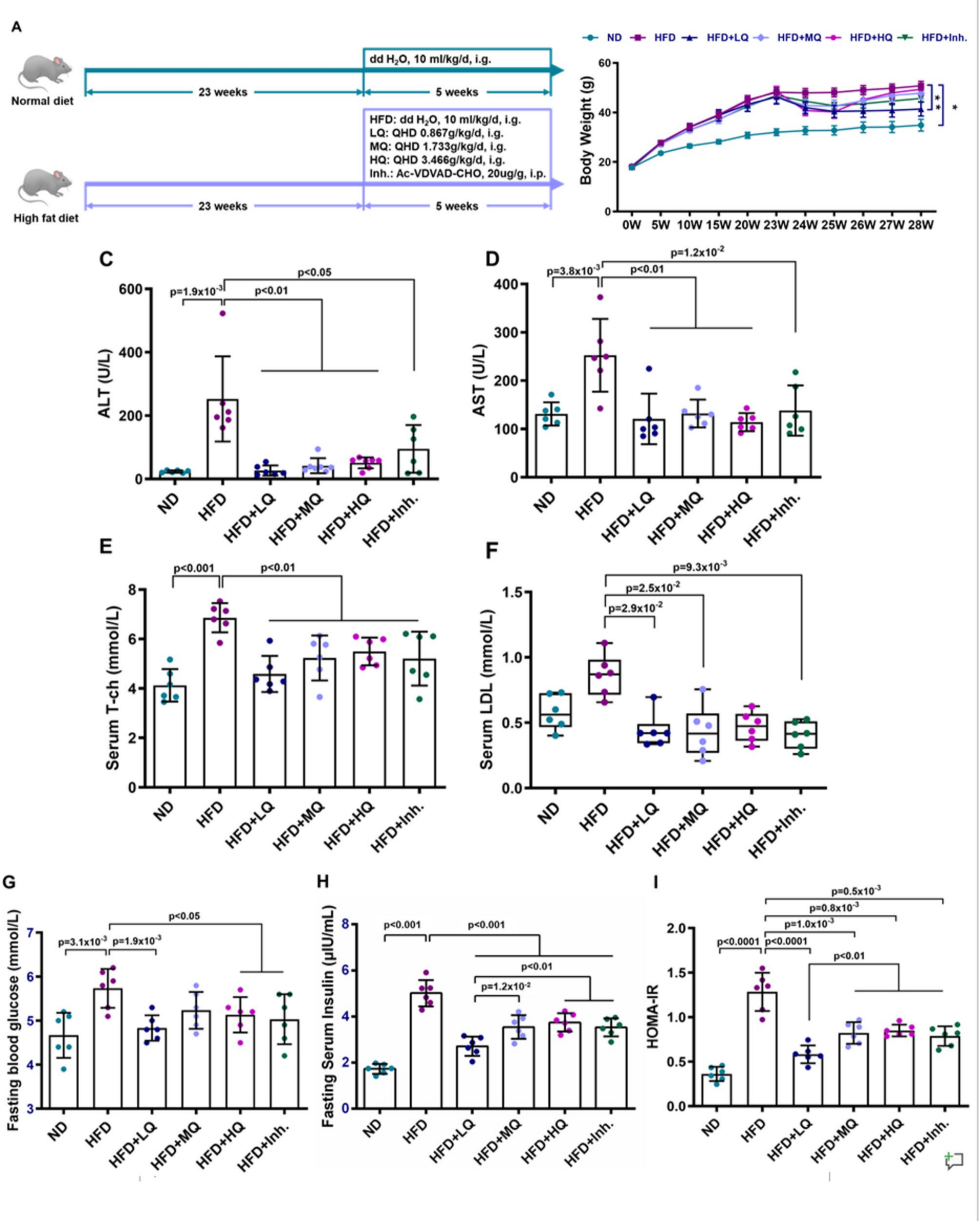
Effects of QHD on body weight, liver enzymes, and metabolic parameters in HFD-induced MASLD mice. (**A**) Schematic representation of the experimental design. C57BL/6J mice were fed either a normal diet (ND) or a high-fat diet (HFD) for 23 weeks, followed by 5 weeks of treatment. Treatment groups included low-dose QHD (LQ, 0.867 g/kg), medium-dose QHD (MQ, 1.733 g/kg), high-dose QHD (HQ, 3.466 g/kg), and the caspase-2 inhibitor Ac-VDVAD-CHO (Inh, 20 µg/g). (**B**) Body weight changes during the experimental period. (**C**–**I**) Biochemical parameters after treatment: (**C**) serum alanine aminotransferase (ALT), (**D**) aspartate aminotransferase (AST), (**E**) total cholesterol (T-Ch), (**F**) low-density lipoprotein cholesterol (LDL-C), (**G**) fasting blood glucose, (**H**) fasting serum insulin, and (**I**) HOMA-IR index. Data are presented as mean ± SD (C, D, E, G, H, I) or median (IQR) (**F**). Statistical significance was determined using one-way ANOVA followed by Tukey’s post hoc test (C, D, E, G, H, I) or Kruskal–Wallis test (**F**). ^*^p < 0.05, ^**^p < 0.01 vs. indicated groups

Thirty-six male C57BL/6J mice, weighing 17 ~ 20 g, were purchased from Shanghai SLAC Laboratory Animal Co., Ltd., and were housed and observed in the SPF-grade animal facility at the Experimental Animal Center of Shanghai University of Traditional Chinese Medicine with free access to food and water. The normal diet group (ND, *n* = 6) was fed a normal diet (10% of calories from fat, 3.85 kcal/g, purchased from Research Diets, New Brunswick). The remaining mice (*n* = 30) were fed a high-fat diet (60% of calories from fat, 5.24 kcal/g, purchased from Research Diets, New Brunswick) for 23 weeks to establish the MASLD model. Afterward, they were divided into the following groups: model group (HFD, *n* = 6), model + QHD low-dose group (HFD + LQ, *n* = 6), model + QHD medium-dose group (HFD + MQ, *n* = 6), model + QHD high-dose group (HFD + HQ, *n* = 6), and model + Caspase-2 inhibitor group (HFD + Inh., *n* = 6). The model was continued, and drug interventions were performed for 5 weeks. The experiment ended at week 28 (Fig. 1A).

Mice in the ND and HFD groups were administered drinking water via gavage at a dose of 10 ml/kg/day. The HFD + LQ group was gavaged with 0.867 g/kg body weight of QHD daily, the HFD + MQ group with 1.733 g/kg body weight of QHD daily, and the HFD + HQ group with 3.466 g/kg body weight of QHD daily. The gavage volume was standardized to 10 ml/kg body weight. The HFD + Inh. group received an intraperitoneal injection of Caspase-2 inhibitor at a dose of 20 µg/g body weight daily. The protocols for animal studies were reviewed and approved by the Animal Studies Ethics Committee of Shanghai University of Traditional Chinese Medicine (permit number: PZSHUTCM211129028).

### Detection of serum biochemical indicators

After the mice were fasted for 12 h, fasting blood glucose was measured using a blood glucose meter (Omron Healthcare Co., Ltd., model: HEA-230). Blood was collected from the eye and allowed to stand at room temperature. After 2 h, the blood was centrifuged (3000 rpm, 10 min), and the supernatant was collected, aliquoted, and stored at -70 °C. After blood collection, the liver and other organ contents were harvested and stored in EP tubes at -70 °C.

Serum alanine aminotransferase (ALT), aspartate aminotransferase (AST), total cholesterol (T-ch), and low-density lipoprotein (LDL) levels were measured using an automatic biochemical analyzer (Shenzhen Leidu Life Sciences Co., Ltd., model: Chemray 240). ALT was measured using the alanine substrate method, and AST was measured using the aspartate substrate method, with a primary wavelength of 340 nm and a secondary wavelength of 405 nm. The test sample volume was 20 µL. T-ch was measured using the CHOD-PAP method, with a primary wavelength of 510 nm and a secondary wavelength of 670 nm, and the test sample volume was 3 µL. LDL was measured using the direct method - surfactant removal method - with a primary wavelength of 546 nm and a secondary wavelength of 670 nm, and the test sample volume was 4.5 µL. Serum insulin was determined using an ELISA kit (Crystal Chem, product number: 90080) according to the manufacturer’s instructions.

### Measurement of liver tissue triglycerides (TG) and free fatty acids (FFA)

Approximately 100 mg of liver tissue was weighed and placed in an EP tube containing acetone and anhydrous ethanol, followed by the addition of small magnetic beads. The mixture was then placed in a homogenizer (homogenizer parameters: 65 Hz, 60 s). After homogenization, the sample was stored overnight at 4 °C. Following overnight incubation, the liver tissue homogenate was centrifuged at room temperature (3000 rpm, 15 min), and the supernatant was carefully collected into an EP tube for further analysis. Liver tissue TG were measured using a colorimetric assay kit. FFA in liver tissue were measured using a colorimetric assay kit.

### Histopathological examination

#### Hematoxylin-eosin (HE) staining

Liver tissue specimens were fixed in 4% paraformaldehyde for 24 h, followed by dehydration and paraffin embedding. Sections were cut at 4 μm thickness, baked onto slides, and dewaxed through a graded series of xylene and ethanol solutions. After rehydration in distilled water, sections were subjected to HE staining. Subsequently, stained sections were dehydrated in absolute ethanol, cleared in xylene, and mounted with neutral resin. Histological morphology was observed under an optical microscope. Two fields per section were randomly selected and imaged at 400× magnification.

#### Oil red O staining of liver tissue

Liver tissue specimens were fixed in 4% paraformaldehyde for 24 h, followed by cryoprotection through graded sucrose solutions (15%/30%) over 3 days. Tissues were embedded in OCT compound and cryosectioned at 20 μm thickness. Sections were stained with Oil Red O working solution for 10 min, then differentiated in 70% ethanol to remove excess dye. After rinsing under running tap water for 1 min, counterstaining was performed with hematoxylin for 2 min followed by a 1-minute tap water rinse. Sections were mounted with glycerol gelatin and scanned using a whole-slide scanner. Histopathological assessment was conducted under an optical microscope, with five random fields per section imaged at 400× magnification.

### Quantitative real-time PCR

Primers required for the experiment were pre - designed and synthesized, and synthesized at Eurofins MWG Operon AG. Total RNA was isolated using an RNA extraction kit (Cat# 9767, purchased from TaKaRa Bio Inc., Japan). Before reverse transcription, the concentration of total RNA was measured using a spectrophotometer, and samples with too low concentration were excluded. cDNA was prepared according to the steps required by the reverse transcription kit (#K1622, purchased from Thermo Fisher Scientific). According to the PCR instructions (RR420A, purchased from TaKaRa Bio Inc., Japan) and the total number of samples, the total amount of each reagent needed was calculated and then the samples were added. The PCR 96 - well plate with 20 µl mixture added was placed in the PCR machine (iCycler IQ System, BioRad Laboratories), and the Comparative Cr mode was selected for detection, with GAPDH as the internal reference to calculate the relative expression of each gene. The primer sequence of Caspase-2 is Caspase-2 – F *ATTCAGCACGTACTCCCACC*, Caspase-2 - R *AGCCTTTCCAGCCTCGAGTA*.

### Western blot

Liver tissue protein lysates were prepared using RIPA lysis buffer supplemented with Complete Mini protease inhibitor cocktail and PMSF (phenylmethylsulfonyl fluoride). Briefly, 100 mg of liver tissue was weighed and placed into pre-prepared RIPA lysis buffer. Tissues were then homogenized to create a uniform suspension. The homogenate was incubated for 2 h at room temperature (25 °C), then centrifuged at 12,000 rpm (approximately 15,300 g) at 4 °C for 20 min. The supernatant, containing the protein lysate, was collected and stored. Nuclear proteins from all mouse liver tissue samples were extracted using a nucleoplasmic separation kit (P0028, Beyotime Biotechnology) according to the manufacturer’s instructions, facilitating the isolation of nuclear fractions.

Protein concentrations were determined using the BCA protein assay kit (P0012, Beyotime Biotechnology) following the manufacturer’s protocol. Equal amounts of protein were separated by 10% SDS-PAGE (sodium dodecyl sulfate-polyacrylamide gel electrophoresis). Separated proteins were then transferred to PVDF (polyvinylidene difluoride) membranes for subsequent immunoblotting analysis .

Following transfer, PVDF membranes were blocked with blocking buffer for 1 h at room temperature to minimize non-specific antibody binding. After blocking, the membranes were incubated with primary antibodies against Caspase-2, SREBP1 (sterol regulatory element-binding protein 1), S1P (sphingosine-1-phosphate), ACC1 (acetyl-CoA carboxylase 1), and SCD1 (stearoyl-CoA desaturase 1) overnight at 4 °C on a rocking platform.

After primary antibody incubation, membranes were washed three times with TBST (Tris-buffered saline with Tween 20) to remove unbound antibody. Membranes were then incubated with appropriate secondary antibodies, diluted 1:5000 in TBST, for 1 h at room temperature. Infrared imaging was performed to visualize protein bands. Protein band density was quantified using electrophoresis image analysis software. The density integration values were calculated, and data were expressed as a ratio of the target band to the corresponding loading control (either GAPDH for total protein or LaminB1 for nuclear protein).

### Caspase-2 activity detection

Cryopreserved liver tissue samples (100 mg) were placed in individual microcentrifuge tubes. Each tube received 1 mL of RIPA lysis buffer and 2–3 grinding beads. Tissues were then homogenized for 2 min using a cryogenic grinder. Following removal of the grinding beads, samples were incubated on ice for 20 min and subsequently centrifuged at 12,000 rpm for 10 min at 4 °C. The supernatant was collected and transferred to a fresh 1.5 mL microcentrifuge tube. Caspase-2 levels were then quantified using a commercially available ELISA kit, following the manufacturer’s instructions.

### The statistical methods

Statistical analyses were performed in GraphPad Prism 6.0. Data are summarized as mean ± SD when distributional assumptions were reasonably met and as median when they were not. For comparisons among *n* ≥ 3 groups, we used one-way ANOVA followed by Tukey’s post-hoc test under normality/variance assumptions; otherwise we used the Kruskal-Wallis test followed by Dunn’s multiple-comparisons test with multiplicity-adjusted *p*-values. Given the small sample size, when normality was uncertain we prioritized non-parametric analyses. Individual data points are shown in all graphs. Statistical significance was defined as *p* < 0.05.

## Results

### Low, medium, and high doses of QHD ameliorate metabolic phenotypes in MASLD mouse model

During the experimental period, mice in the normal diet (ND) group exhibited good health status, were responsive, possessed smooth and shiny fur coats, had normal stool consistency, and maintained moderately humid bedding within their cages. In contrast to the normal group, mice in the high-fat diet model (HFD) group displayed accelerated body weight gain, reduced responsiveness in later stages, greasier fur coats with noticeable hair loss. Following the initiation of drug intervention at week 23, all treatment groups exhibited a significant decrease in body weight for two weeks. Thereafter, body weight gradually increased from week 3 onwards, with the Low-Dose QHD group showing the slowest rate of increase. Upon comparing the final body weights at the end of the experiment, the model group showed a significant increase compared to the normal group. The body weight of the Low-Dose QHD group was significantly lower than that of the model group. No statistically significant differences in body weight were observed between the model group and the other treatment groups (Fig. 1B).

In improving liver function, QHD also demonstrated remarkable efficacy. Low-, Medium-, and High-Dose QHD consistently reduced serum levels of ALT and AST in model mice. The Caspase-2 inhibitor also achieved statistically significant reductions in ALT and AST levels (Fig. 1C, D).

Experimental results demonstrated that QHD significantly reduced serum T-ch levels. Compared with the normal group, the model group exhibited significantly elevated serum T-ch levels. QHD treatment at low and mid doses significantly reduced LDL levels compared to the HFD group (*P* < 0.05), while the high-dose group did not reach statistical significance. (Fig. 1E, F).

Compared with the ND group, HFD group mice exhibited significantly elevated fasting blood glucose (FBG), fasting serum insulin (FSI), and homeostasis model assessment of insulin resistance (HOMA-IR). Relative to the HFD group, both Low-Dose and High-Dose QHD groups demonstrated significant reductions in FBG. All treatment groups showed significantly decreased FSI and HOMA-IR levels. Notably, the Low-Dose QHD group demonstrated superior efficacy in improving FSI and HOMA-IR, with values significantly lower than those of the other treatment groups (Fig. 1G, H, I).

These findings indicate that Low-Dose QHD comprehensively optimized metabolic parameters in MASLD model mice.

### Amelioration of hepatic lipid deposition in MASLD model mice by low-, medium-, and high-dose QHD

**Fig. 2:**
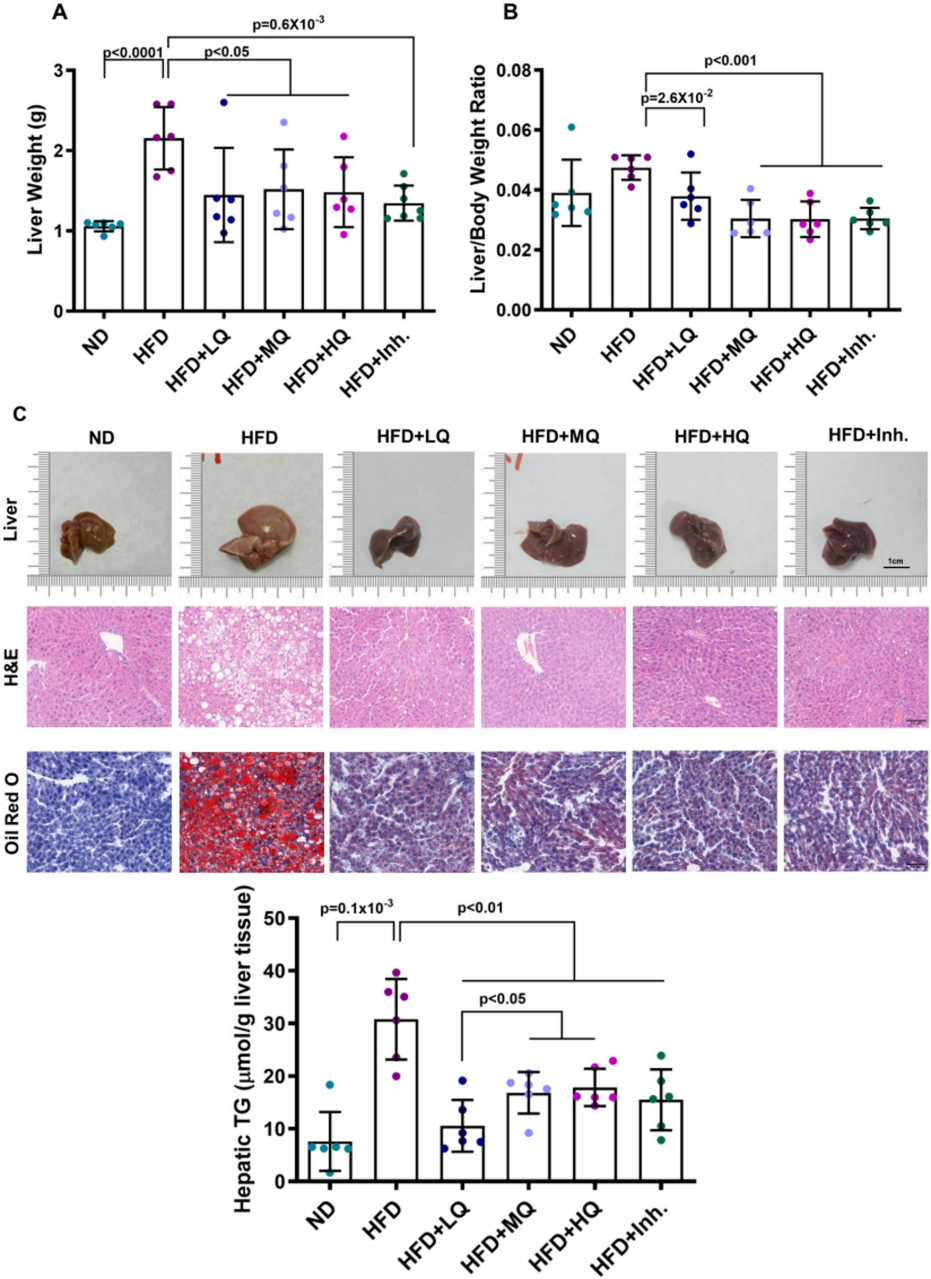
Effects of QHD on liver morphology, histology, and hepatic triglyceride accumulation in HFD-induced MASLD mice. (**A**) Liver weight and (**B**) liver-to-body weight ratio in different treatment groups. (**C**) Representative liver morphology (upper row), hematoxylin and eosin (**H**&**E**) staining (middle row), and Oil Red O staining (bottom row) from each group. Scale bar = 20 μm (H&E, Oil Red O). (**D**) Hepatic triglyceride (TG) levels measured in liver tissue. Data are presented as mean ± SD. Statistical significance was determined using one-way ANOVA followed by Tukey’s post hoc test

The livers of ND group and treatment group mice exhibited bright red coloration, firm texture, and sharp edges. In contrast, HFD group livers displayed marked enlargement, pale color, greasy appearance, and rounded edges (Fig. 2C). Hepatic wet weight was significantly elevated in the HFD group compared to the ND group, while all treatment groups showed significant reductions in both hepatic wet weight and liver-to-body weight ratio relative to the HFD group (Figs. 2A and B). Histopathological analysis (H&E and Oil Red O staining) revealed substantially attenuated hepatocellular steatosis in all treatment groups compared to the model group (Fig. 2C).

Compared with the ND group, hepatic TG and FFA levels were significantly elevated in the HFD group, confirming successful model establishment. All treatment groups exhibited significantly reduced hepatic TG and FFA levels versus the HFD group. Notably, the Low-Dose QHD group demonstrated more pronounced TG reduction compared to other treatment groups (Figs. 2D and E).

### High-dose QHD suppresses Caspase2/SREBP1 pathway expression in MASLD model mice

**Fig. 3:**
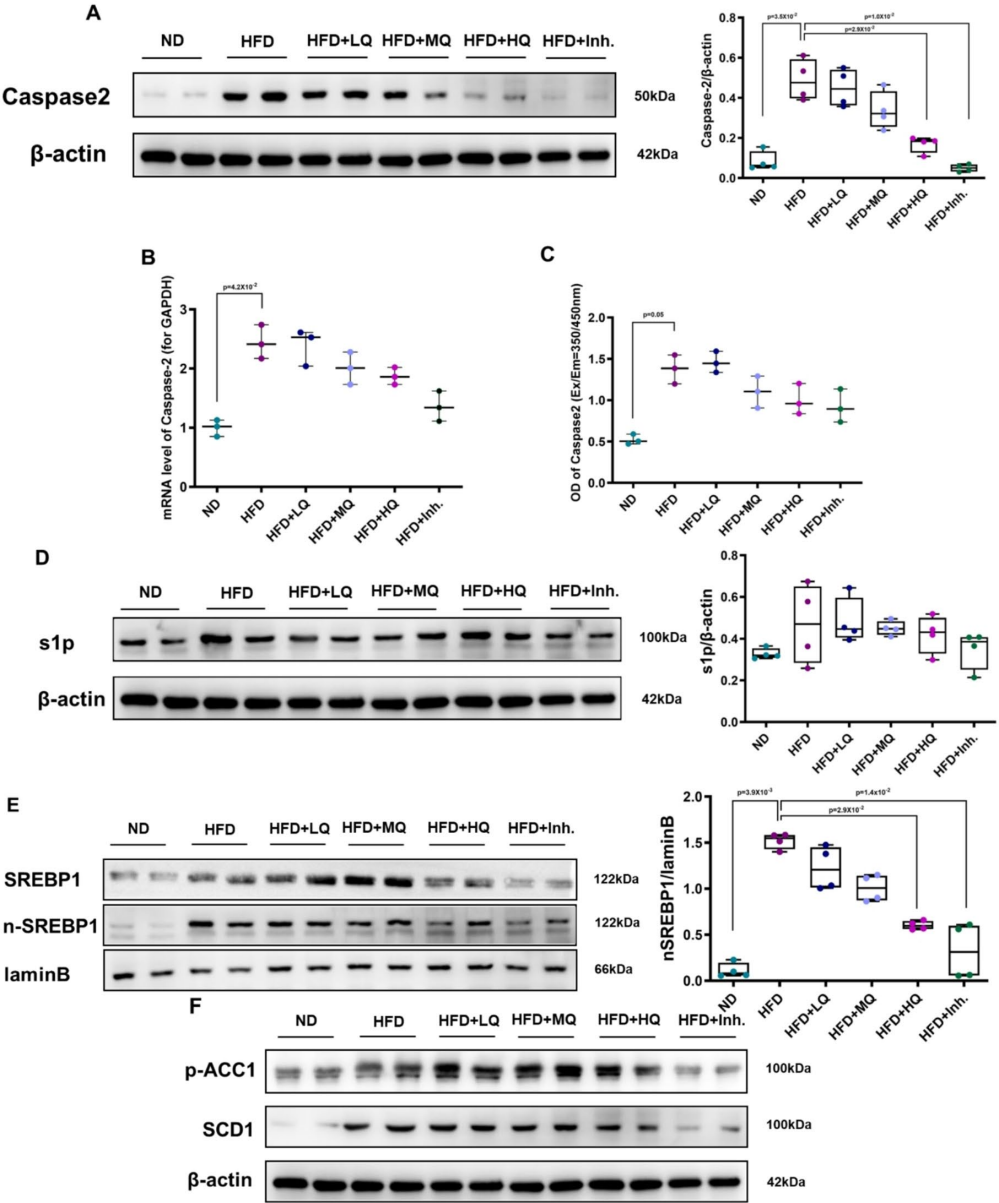
Effects of QHD on Caspase-2/SREBP-1 signaling pathway in HFD-induced MASLD mice. (**A**) Representative Western blot and quantification of Caspase-2 protein expression in liver tissues. (**B**) Relative mRNA expression of Caspase-2 measured by qPCR. (**C**) Caspase-2 enzymatic activity assessed by colorimetric assay. (**D**) Western blot and quantification of S1P protein expression. (**E**) Western blot of total SREBP-1 and nuclear SREBP-1 (n-SREBP-1), with lamin B as nuclear loading control. (**F**) Western blot of downstream lipid metabolism markers p-ACC1 and SCD1. Statistical significance was determined using Kruskal-Wallis test with Dunn’s post hoc test

Compared to the ND group, hepatic Caspase-2 and n-SREBP1 protein expression were significantly elevated in the HFD group. Conversely, the High-Dose QHD group exhibited markedly reduced expression of both proteins relative to the HFD group (Figs. 3A, E). Correspondingly, hepatic Caspase-2 mRNA levels and Caspase-2 activity were significantly higher in the HFD group than in the ND group. All QHD treatment groups (low-, medium-, and high-dose) demonstrated a dose-dependent reduction in these parameters (Figs. 3B, C), with the most pronounced decrease observed in the High-Dose group. S1P expression, however, did not show a consistent trend among groups (Fig. 3D). The protein expression of p-ACC1 and SCD-1, the downstream lipid synthesis-related genes of SREBP1 (Fig. 3F), was not further counted because of the small sample size, but grey-scale measurement showed that the grey-scale values still showed a dose-dependent decreasing trend, with the lowest value in the High-Dose group.

## Discussion

Our findings suggest a dose-dependent but complementary pattern of QHD’s actions on MASLD. The 28-week HFD-induced MASLD model employed in this study offers a robust framework for assessing dose-related effects. Prolonged metabolic stress in this model markedly activates Caspase-2, thereby enhancing the visibility of QHD’s regulatory actions [8]. Notably, this chronic paradigm resembles clinical TCM treatment durations (typically ≥ 6 months), highlighting the potential relevance of QHD for long-term interventions.

Experimental results demonstrated that at lower doses QHD more prominently improved metabolic parameters such as hepatic TG, FFA, and HOMA-IR. This metabolic phenotype improvement aligns with prior findings from our research team. Previous studies have established that low-dose QHD activates AMPK/PGC-1α signaling to induce adipose tissue browning, elevates CPT-1 A expression, and thereby enhances fatty acid β-oxidation. Simultaneously, it corrects branched-chain amino acid (BCAA) metabolic disorders and improves mitochondrial function [15].

**Fig. 4:**
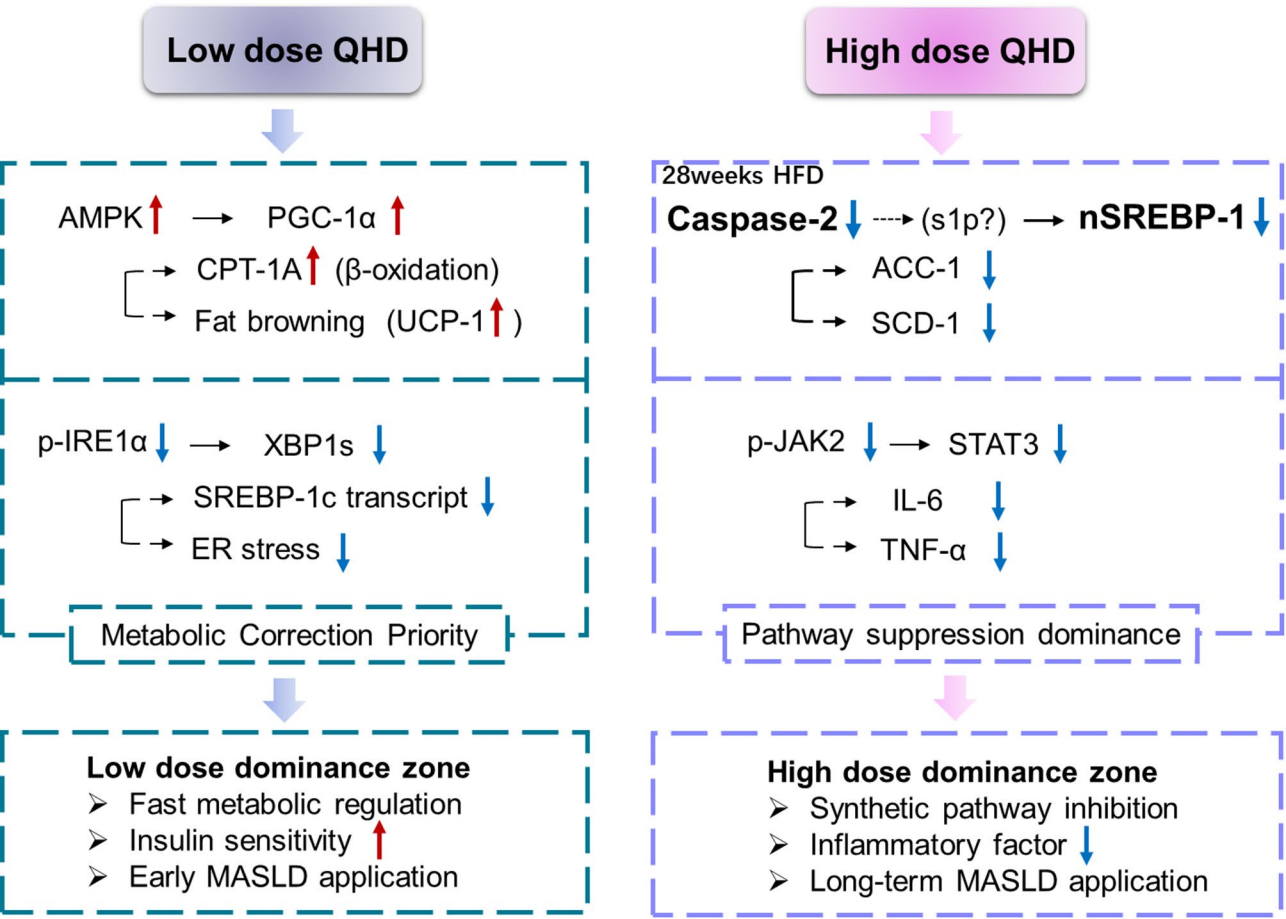
Dose-dependent dual-mode mechanism of QHD in MASLD. Left (Low-dose QHD): QHD activates AMPK, leading to increased PGC-1α and CPT-1 A and promotion of adipose browning (UCP-1↑). In parallel, reduced IRE1α phosphorylation lowers XBP1s and decreases SREBP-1c transcription, prioritizing metabolic correction. Right (High-dose QHD): Under chronic HFD stress, high-dose QHD suppresses Caspase-2, thereby reducing nuclear SREBP-1 and downstream lipogenic enzymes (ACC-1, SCD-1). In addition, inhibition of the JAK2/STAT3 axis lowers IL-6 and TNF-α. These combined effects yield overall ER-stress attenuation, representing pathway-suppression dominance. Bottom panels summarize the clinical implications: low dose favors rapid metabolic regulation and insulin sensitivity (early MASLD), while high dose emphasizes inhibition of synthetic and inflammatory pathways for long-term management

The mechanistic diagram in Fig. 4 illustrates the dose-dependent actions of QHD on MASLD, distinguishing the metabolic correction at low doses from the pathway suppression at high doses. Notably, inhibition of the IRE1α–XBP1s branch was observed across all QHD dose groups. This universal effect leads to decreased SREBP-1c transcription and contributes to improved lipid metabolism. In our framework, this pathway is shown in the low-dose panel because its functional consequence is most directly linked to metabolic correction and early improvement of insulin sensitivity, which are clinically relevant in the early stages of MASLD. In the low-dose dominance zone, AMPK activation further upregulates PGC-1α and CPT-1 A, enhancing fatty acid β-oxidation and promoting adipose tissue browning. Together, these mechanisms emphasize the role of low-dose QHD in rapidly correcting metabolic disturbances.

At higher doses, QHD was associated with more pronounced suppression of ER stress–related signaling, including reduced Caspase-2 and nuclear SREBP-1 expression, along with modulation of inflammatory pathways. While IRE1α inhibition remains present, the distinguishing features of high-dose treatment are the downregulation of Caspase-2 and nuclear SREBP-1, suppression of JAK2/STAT3 signaling, and consequent reduction of pro-inflammatory cytokines (IL-6, TNF-α). These combined effects result in an overall attenuation of ER stress, reflecting a threshold-dependent pharmacodynamic effect under chronic metabolic stress. This dual profile explains why IRE1α inhibition appears in both dose settings experimentally, but is emphasized in the low-dose panel of Fig. 4 as part of metabolic correction, while high-dose QHD achieves broader suppression of pathogenic signaling pathways. Interestingly, S1P expression did not display a consistent trend across groups, even when Caspase-2 was markedly suppressed. This variability may reflect the complex regulation of S1P under chronic metabolic stress, as its expression is influenced by multiple pathways beyond Caspase-2, such as IRE1α-XBP1 signaling and ceramide metabolism [24]– [25]. In addition, the labile nature of S1P protein during extraction and detection may contribute to experimental variability. Taken together, these results support a hierarchical modulation model, where metabolic correction and pathway suppression represent dose-related yet complementary effects of QHD. This framework emphasizes the potential advantage of multi-component herbal formulations, which dynamically adjust ER stress and metabolic networks rather than acting on a single target. Importantly, we acknowledge that the causal links remain correlative, and future studies incorporating genetic manipulation and bioactive compound identification are required to verify direct molecular targets.

This dose-dependent phenomenon likely reflects a threshold pharmacodynamic requirement for sustained target inhibition in chronic models. This observation is consistent with the multicomponent pharmacokinetic profile of herbal formulations. Key bioactive constituents in QHD (e.g., curcumin, resveratrol) undergo rapid absorption, while at lower doses, AMPK (as a master cellular energy sensor) is preferentially activated by these small molecules. This mechanism accelerates fatty acid oxidation and promotes adipose tissue browning, facilitating rapid correction of metabolic dysregulation [26, 27].

In this study, we propose a mechanistic framework by which the Traditional Chinese Medicine compound QHD ameliorates MASLD through dose-dependent modulation of distinct ER stress branch pathways. In contrast to the Caspase-2–centric model proposed by Kim et al. [6], our findings suggest that the therapeutic advantage of QHD may lie in its hierarchical regulation of the ER stress network. High-dose QHD predominantly targets the downstream effector Caspase-2 to mitigate lipotoxicity, while low-dose QHD more strongly reflects inhibition of IRE1α phosphorylation, thereby supporting metabolic homeostasis [17]. This differentiated modulation strategy may offer advantages over single-target inhibitors. For instance, it avoids disrupting essential apoptotic functions, which can occur with Caspase-2 knockout. Moreover, this approach aligns with the updated MASLD definition, which emphasizes metabolic dysfunction.

For guiding clinical translation, this multi-tiered modulation strategy resonates profoundly with the Traditional Chinese Medicine (TCM) principle of “sovereign, minister, assistant, and courier” herbs. Specifically, high-dose components act as “sovereign herbs” to directly target core pathological nodes, while low-dose components function as “minister herbs” to modulate the metabolic microenvironment [28–30]. This alignment provides a novel framework for personalized MASLD treatment. For instance, in early-stage MASLD dominated by predominant metabolic dysregulation, low-dose QHD can be prioritized to correct metabolic disturbances, rapidly alleviate symptoms, and reduce patient medication burden. In contrast, for patients with mid-to-late-stage MASLD characterized by heightened de novo lipogenesis (DNL), where intervention may be delayed, high-dose QHD can be administered to specifically inhibit key pathways such as Caspase-2/SREBP1 and JAK2/STAT3. This effectively blocks the expression of DNL-associated genes and rapidly suppresses inflammatory responses, thereby preventing further escalation of ER stress.

In clinical translation, such a dose-dependent strategy may be implemented as staged treatment planning. For early-stage MASLD patients dominated by metabolic dysregulation, a low-dose QHD regimen administered for 8 ~ 12 weeks may be sufficient to restore metabolic homeostasis, improve insulin sensitivity, and reduce hepatic fat accumulation. In contrast, for mid-to-late-stage MASLD patients characterized by heightened de novo lipogenesis and inflammation, a higher-dose regimen sustained for 12 ~ 24 weeks may be required to achieve significant suppression of lipogenic and inflammatory pathways. These proposed timeframes are consistent with the 24-week treatment courses that have already been employed in multicenter randomized controlled trials (RCTs) of QHD, but our data suggest that more flexible and stage-specific dosing strategies may further optimize clinical outcomes. To verify this hypothesis, future prospective clinical studies will be designed to compare dose-escalation regimens (e.g., stepwise escalation from low to high dose) with fixed-dose protocols, thereby providing stronger evidence for personalized QHD therapy in MASLD.

While this study provides valuable insights into the dose-dependent effects of QHD in MASLD, several limitations must be addressed. One key limitation is the small sample size used in some protein expression analyses (e.g., p-ACC1, SCD-1), which may limit the statistical power of these results. The sample size was constrained by experimental design considerations and available resources. We acknowledge that expanding the sample size in future studies would allow for more robust quantification and verification of these findings. Additionally, the duration of the intervention in this study was limited to 5 weeks, and extending the treatment duration could provide more comprehensive insights into the long-term effects of QHD on metabolic and hepatic parameters. Furthermore, this study primarily focused on steatosis and metabolic parameters; consequently, fibrosis progression was not assessed using specific staining techniques such as Sirius Red or α-SMA. Future work should include detailed fibrosis evaluation to fully elucidate QHD’s potential in mitigating advanced MASLD progression. Future work should also aim to include larger sample sizes for Caspase-2 activity assays and further explore the molecular mechanisms underlying QHD’s action, including the identification of active bioactive compounds using techniques like HPLC-QTOF/MS. Moreover, employing membrane localization assays for S1P receptor dynamics could provide more precise insights into S1P-related signaling, an area that was beyond the scope of this study due to funding constraints. Furthermore, the variability in S1P expression observed in the study might have been influenced by factors such as protein degradation or detection sensitivity. This fluctuation in S1P expression will be further investigated in future studies with additional controls to better understand its role and dynamics in MASLD.

## Conclusion

Our study demonstrates that QHD exerts complementary, dose-dependent effects in MASLD: low doses predominantly improve lipid metabolism and insulin sensitivity, whereas high doses are associated with stronger suppression of Caspase-2/SREBP-1 and inflammatory signaling, highlighting QHD’s broader regulatory impact on ER stress. These findings support a hierarchical modulation model in which metabolic correction and pathway suppression act as complementary mechanisms, reflecting the pharmacodynamic complexity of multi-component herbal formulations. Translationally, this dual pattern provides a rationale for stage-specific dosing strategies in MASLD, consistent with the traditional Chinese medicine principle of “*sovereign*–*minister*–*assistant*–*courier*”.

## Supplementary Information

Below is the link to the electronic supplementary material.

Supplementary Material 1

## Data Availability

No datasets were generated or analysed during the current study.
